# Effects of Preservative-free 3% Diquafosol in Patients with Pre-existing Dry Eye Disease after Cataract Surgery: A Randomized Clinical Trial

**DOI:** 10.1038/s41598-019-49159-0

**Published:** 2019-09-02

**Authors:** Ikhyun Jun, Seonghee Choi, Geun Young Lee, Young Joon Choi, Hyung Keun Lee, Eung Kweon Kim, Kyoung Yul Seo, Tae-im Kim

**Affiliations:** 1 The Institute of Vision Research, Department of Ophthalmology, Yonsei University College of Medicine, 50-1 Yonsei-ro, Seodaemungu, Seoul 03722 Korea; 2 Corneal Dystrophy Research Institute, Department of Ophthalmology, Yonsei University College of Medicine, 50-1 Yonsei-ro, Seodaemungu, Seoul 03722 Korea

**Keywords:** Drug therapy, Eye manifestations

## Abstract

Dry eye disease (DED) after cataract surgery has become a critical concern, and various therapeutic options have been developed. Recently, preservative-free diquafosol ophthalmic solution has been introduced; however, its therapeutic effect on DED after cataract surgery has not been reported. We investigated the efficacy of preservative-free diquafosol in patients with pre-existing DED after cataract surgery. We divided subjects who were diagnosed with DED and scheduled to undergo cataract surgery, into 3 groups (preservative-free diquafosol, group 1; preservative-containing diquafosol, group 2; preservative-free hyaluronate, group 3), and each eye drops was administered 6 times daily after surgery. Tear break up time (TBUT), Ocular Surface Disease Index (OSDI), corneal staining score, lid margin abnormality, and meibum quality improved over time in group 1. Groups 1 and 2 had significantly superior TBUT, meibomian gland dysfunction grade, and meibomian gland expressibility throughout the study period than group 3. Meibum quality of group 1 was significantly better than group 2 at 1 and 3 months after surgery. Preservative-free diquafosol showed better efficacy in treating DED after cataract surgery than preservative-containing diquafosol or preservative-free hyaluronate. Preservative-free diquafosol may serve as a reliable option for the management of patients with pre-existing DED after phacoemulsification.

## Introduction

Today, cataract surgery has become one of the safest and most effective ocular surgical procedures owing to improvements in surgical techniques and instruments. However, a significant number of patients who undergo cataract surgery suffer postoperative discomfort and irritation, pain, dryness, burning sensation, and foreign body sensations^1–3^. Potential cause(s) of dry eye disease (DED) after cataract surgery include microscope-induced thermal and light toxicity, mechanical damage to the corneal tissue, irrigation of the ocular surface, chemical sterilization of the conjunctival sac and eyelid, transection of the corneal nerves by corneal incision, topical anaesthetic use, and use of topical eye drops containing preservatives^4–6^. Moreover, several recent studies have reported that a common cause of postoperative discomfort in these patients is pre-existing DED^6,7^. The deformed ocular surface due to DED after surgery affects optical quality considerably, resulting in decreased patient satisfaction with vision quality. In the era of improved surgical outcomes and extremely elevated patient expectations due to a proven high level of surgical accuracy, DED related postoperative discomfort is unacceptable anymore.

Many options to treat DED after cataract surgery have been developed^8^. Artificial tears are commonly used as a first line management strategy for postoperative DED, with several studies revealing their effectiveness at reducing DED signs and symptoms^9,10^. The postoperative use of cyclosporine 0.05% topical eye drops has led to improvement in dry eye symptoms and visual quality following cataract surgery^11,12^. Recently, diquafosol sodium ophthalmic solution has also been used for the management of DED after cataract surgery^2,5,6,13^. Diquafosol is a dinucleotide derivative and functions as an agonist of the purinergic P2Y_2_ receptor^14^. Diquafosol is known to stimulate not only mucin secretion from goblet cells but also water secretion from conjunctival epithelial cells and the accessory lacrimal glands^14,15^. Studies have shown that diquafosol is very effective in treating and alleviating symptoms of DED after cataract surgery^2,5,6,13^. Furthermore, several studies have shown that topical diquafosol is more effective for managing DED after cataract surgery than artificial tears^5,6^. Preservative-free diquafosol ophthalmic solution has been introduced recently. The use of other eye drops without preservatives has been shown to play an important role in the treatment of DED after cataract surgery with reduced adverse reactions^16^. At present, however, there is no study that has evaluated the therapeutic effect of preservative-free diquafosol ophthalmic solution on DED after cataract surgery. Therefore, in this study, we compared the efficacy of preservative-free diquafosol ophthalmic solution with that of preservative-containing diquafosol and preservative-free sodium hyaluronate ophthalmic solutions, which are widely used in patients with DED after cataract surgery.

## Results

This study included 38 eyes in group 1, 41 eyes in group 2, and 38 eyes in group 3. Table 1 presents the baseline characteristics of the 3 groups of patients. There were no significant differences in laterality, age, or sex among the 3 groups (Table 1). There were no significant differences in preoperative tear break up time (TBUT) (4.6 ± 2.2, 5.0 ± 2.5, and 4.6 ± 1.8 s for groups 1, 2, and 3, respectively) or Ocular Surface Disease Index (OSDI) (22.10 ± 12.93, 23.49 ± 11.38, and 22.30 ± 9.01 for groups 1, 2, and 3, respectively) among the 3 groups. In addition, no significant differences were observed in preoperative corneal staining scores, Schirmer test scores, or meibomian gland dysfunction (MGD) parameters among the 3 groups (Table 1). There was no significant difference in operation time among the 3 groups (P = 0.097).

**Table 1:** Demographic and Other Baseline Characteristics for Each Group of Patients with Dry Eye Before Cataract Surgery.

	Group 1 (38 eyes)	Group 2 (41 eyes)	Group 3 (38 eyes)	*P*-value
Age (y)	67.7 ± 8.3	71.6 ± 9.4	68.0 ± 7.6	0.058
Sex				
Male	13	17	12	0.694
Female	25	24	26	
Eyes				
OD	22	20	19	0.715
OS	16	21	19	
TBUT (s)	4.6 ± 2.2	5.0 ± 2.5	4.6 ± 1.8	0.925
Schirmer test (mm)	15.5 ± 8.6	13.1 ± 8.7	11.5 ± 7.6	0.076
Corneal staining score	0.66 ± 1.07	0.63 ± 0.89	0.84 ± 0.59	0.051
OSDI	22.10 ± 12.94	23.49 ± 11.38	22.30 ± 9.01	0.836
LLT (nm)	80.82 ± 18.77	86.00 ± 19.17	82.25 ± 20.10	0.484
MGD stage	2.61 ± 1.11	2.44 ± 1.18	2.34 ± 1.10	0.407
Lid margin abnormality	2.58 ± 1.00	2.34 ± 1.37	2.03 ± 1.20	0.174
Meibomian gland expressibility	1.84 ± 1.05	1.88 ± 1.01	1.61 ± 0.95	0.327
Meibum quality	12.18 ± 5.92	10.22 ± 5.73	10.16 ± 6.77	0.152
Meibomian gland dropout	1.11 ± 0.73	1.37 ± 1.07	1.47 ± 1.13	0.467

Data are presented as mean ± standard deviation. TBUT = tear breakup time; OSDI = Ocular Surface Disease Index; LLT = lipid layer thickness; MGD = meibomian gland dysfunction. *P*-value indicates the statistical analysis among the three groups.

### Dry eye parameters

**Figure 1:**
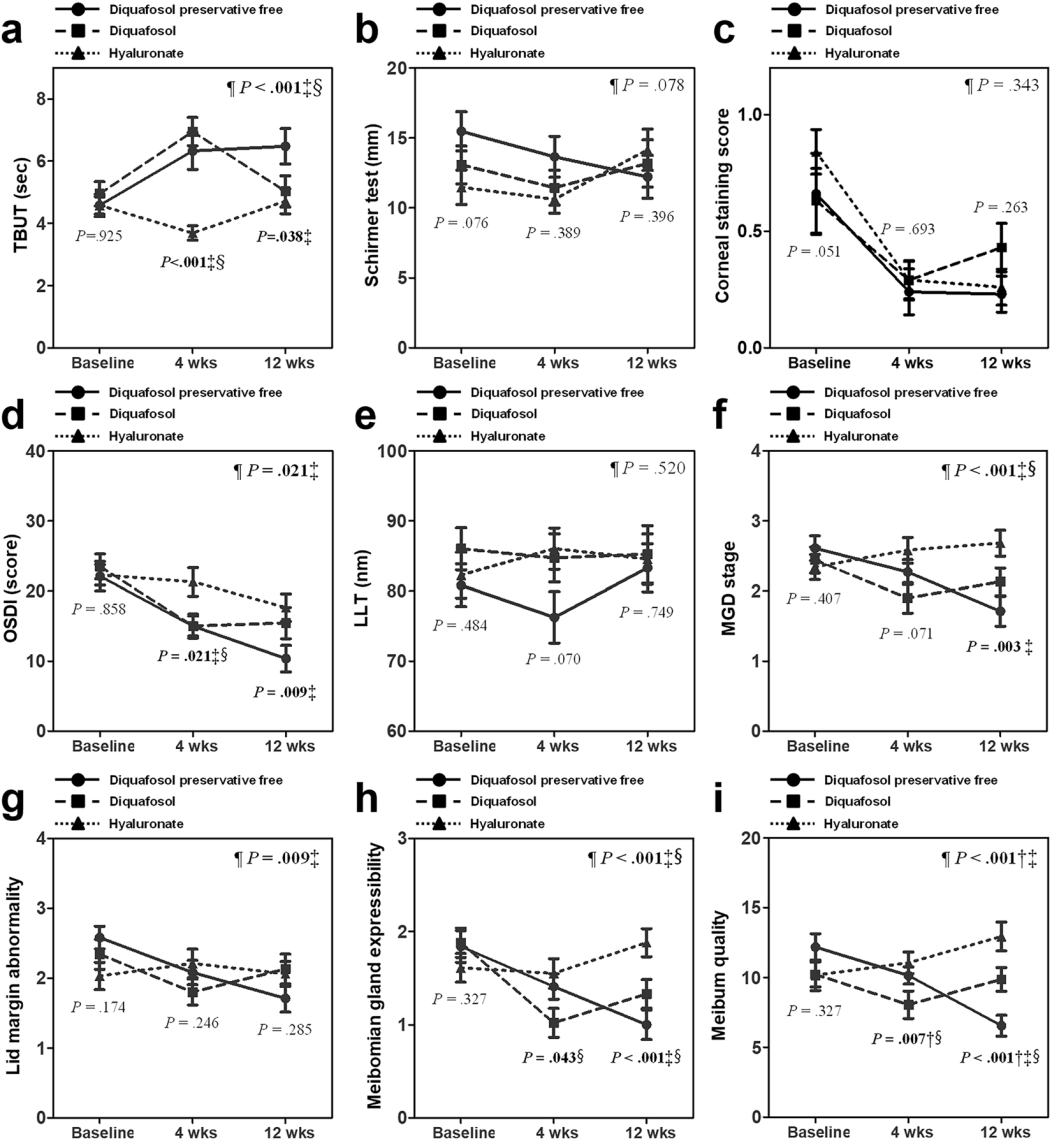
Dry eye changes and meibomian gland dysfunction (MGD) parameters during the treatment period in patients with pre-existing dry eye disease (DED). Mean changes in tear breakup time (TBUT) **(a)**, Schirmer test scores **(b)**, corneal staining scores **(c)**, and Ocular Surface Disease Index (OSDI) **(d)** were used to assess dry eye parameters. Mean changes in lipid layer thickness (LLT) **(e)**, MGD stage **(f)**, lid margin abnormalities **(g)**, meibomian gland expressibility **(h)**, and meibum quality (**i)** were used as MGD parameters. Data are presented as mean values ± standard error of the mean (SEM). *P*-values are presented next to all graphs for each study period, indicating the results of statistical analyses among the 3 groups for that period using analysis of variance; ^¶^*P*-value indicates the overall difference analysis throughout the study period by generalized estimating equation; ^†^indicates a statistically significant difference between groups 1 and 2 per post hoc analysis; ^‡^indicates a statistically significant difference between groups 1 and 3 per post hoc analysis; ^§^indicates a statistically significant difference between groups 2 and 3 per post hoc analysis.

The preoperative and postoperative dry eye parameters for the 3 groups are summarized in Table 2. The TBUT for groups 1 and 2 showed significant improvement from the preoperative values and were significantly longer than that of group 3 at 1 month postoperatively. However, at 3 months after surgery, only group 1 evidenced significant improvements from the preoperative values and had significantly longer TBUT than that of group 3. We also found significant improvements in in TBUT in groups 1 and 2 when compared to group 3 in the overall difference analysis (P < 0.001; Fig. 1a).

**Table 2:** Changes in Dry Eye Parameters and Meibomian Gland Dysfunction Parameters between Preoperative and 4 and 12 Weeks After Cataract Surgery.

	Group 1 (38 eyes)	Group 2 (41 eyes)	Group 3 (38 eyes)	*P*-value
TBUT (sec)				
Preoperative	4.6 ± 2.2	5.0 ± 2.5	4.6 ± 1.8	0.925
4wks	6.3 ± 3.6*	7.0 ± 2.8*	3.7 ± 1.4^‡§^	<**0.001**
12wks	6.5 ± 3.5*	5.0 ± 2.8	4.7 ± 2.3^‡^	**0.038**
Schirmer test (mm)				
Preoperative	15.5 ± 8.6	13.1 ± 8.7	11.5 ± 7.6	0.076
4wks	13.7 ± 8.9	11.4 ± 8.0	10.6 ± 6.1	0.389
12wks	12.2 ± 9.0	13.2 ± 9.3	14.2 ± 8.6	0.396
Corneal staining score				
Preoperative	0.66 ± 1.07	0.63 ± 0.89	0.84 ± 0.59	0.051
4wks	0.24 ± 0.60*	0.29 ± 0.51*	0.29 ± 0.52*	0.693
12wks	0.23 ± 0.43*	0.43 ± 0.57*	0.26 ± 0.45*	0.263
OSDI				
Preoperative	22.10 ± 12.94	23.49 ± 11.38	22.30 ± 9.01	0.836
4wks	14.99 ± 10.42*	15.00 ± 9.19*	21.29 ± 12.57^‡§^	**0.015**
12wks	10.35 ± 10.55*	15.43 ± 12.24*	17.63 ± 11.45* ^‡^	**0.037**
LLT (nm)				
Preoperative	80.82 ± 18.77	86.00 ± 19.17	82.25 ± 20.10	0.484
4wks	76.24 ± 21.32	84.70 ± 21.66	86.03 ± 18.15	0.070
12wks	83.29 ± 19.91	85.23 ± 22.70	84.55 ± 22.12	0.749
MGD stage				
Preoperative	2.61 ± 1.11	2.44 ± 1.18	2.34 ± 1.10	0.407
4wks	2.27 ± 1.07*	1.90 ± 1.39*	2.58 ± 1.13	0.071
12wks	1.71 ± 1.19*	2.13 ± 1.07	2.68 ± 1.07^‡^	**0.003**
Lid margin abnormality				
Preoperative	2.58 ± 1.00	2.34 ± 1.37	2.03 ± 1.20	0.174
4wks	2.08 ± 1.06*	1.80 ± 1.19*	2.21 ± 1.26	0.246
12wks	1.71 ± 1.10*	2.13 ± 1.17	2.06 ± 1.04	0.285
Meibomian gland expressibility				
Preoperative	1.84 ± 1.05	1.88 ± 1.01	1.61 ± 0.95	0.327
4wks	1.41 ± 0.83	1.02 ± 0.99*	1.55 ± 0.98^§^	**0.043**
12wks	1.00 ± 0.89*	1.33 ± 0.84*	1.88 ± 0.88^‡§^	<**0.001**
Meibum quality				
Preoperative	12.18 ± 5.92	10.22 ± 5.73	10.16 ± 6.77	0.327
4wks	10.14 ± 3.74*	8.05 ± 6.29* ^†^	11.05 ± 4.75^§^	**0.007**
12wks	6.55 ± 4.15*	9.86 ± 4.54^†^	12.94 ± 6.02* ^‡§^	<**0.001**
Meibomian gland dropout				
Preoperative	1.11 ± 0.73	1.37 ± 1.07	1.47 ± 1.13	0.467
4wks	1.09 ± 0.75	1.34 ± 0.99	1.42 ± 1.18	0.547
12wks	1.06 ± 0.74	1.44 ± 1.01	1.50 ± 1.16	0.234

Data are presented as means ± standard deviation. TBUT = tear breakup time; OSDI = Ocular Surface Disease Index; LLT = lipid layer thickness; MGD = meibomian gland dysfunction. *P*-value indicates the statistical analysis among the three groups. ^*^Indicates a statistically significant compared with preoperative value; ^†^indicates a statistically significant difference between groups A and B in post hoc analysis; ^‡^indicates a statistically significant difference between groups A and C in post hoc analysis; ^§^indicates a statistically significant difference between groups B and C in post hoc analysis.

OSDI scores were significantly reduced in groups 1 and 2 after surgery across all study periods. There were significant differences between groups 1 and 3 and between groups 2 and 3 in OSDI scores at 1 month after surgery. At 3 months postoperatively, OSDI scores for group 3 were significantly reduced below the preoperative value. Given this, there was no significant difference noted between groups 2 and 3. However, at 3 months after surgery, the reduction in OSDI scores of group 1 was higher than that of the other 2 groups and a significant difference in OSDI scores between groups 1 and 3 persisted. In the overall difference analysis, a significant difference was also observed between groups 1 and 3 (Fig. 1d; P = 0.021).

Corneal staining scores for all groups were significantly better than the preoperative values across all study periods; however, there were no significant differences among the 3 groups. Schirmer test results remained unchanged from the preoperative values and there were no differences among the 3 groups during the entire study period. There were no significant overall differences among the 3 groups with respect to corneal staining scores or Schirmer test scores.

### Meibomian gland dysfunction parameters

The parameters for MGD are summarized in Table 2. At 1 month after surgery, the MGD stage was significantly reduced in group 2 from that before surgery, although there was no significant difference among the 3 groups. However, at 3 months postoperatively, only group 1 showed significant improvement from the preoperative value, with significantly better MGD stage results than group 3. In the overall difference analysis, MGD stage was significantly better in group 1 and group 2 when compared with group 3 (Fig. 1; P < 0.001).

At 3 months after surgery, meibomian gland expressibility of groups 1 and 2 significantly improved from the preoperative condition and showed significant differences when compared with group 3. Similarly, there were significant differences between groups 1 and 3 and between groups 2 and 3, as revealed by overall difference analyses (Fig. 1; P < 0.001). The grade of lid margin abnormality and meibum quality in groups 1 and 2 were significantly improved from the preoperative conditions at 1 month postoperatively; however, only group 1 experienced significant improvements at 3 months after surgery. Although there were no significant differences among the 3 groups in lid margin abnormality, overall difference analysis showed significant improvement in lid margin abnormality of group 1 when compared with group 3. Meibum quality of group 1 demonstrated statistically significant improvements when compared with group 2 or group 3 in overall difference analysis (Fig. 1; P < 0.001). The lipid layer thickness (LLT) was unchanged after surgery, and there were no differences noted among the 3 groups across the entire study period.

### Higher-order aberrations

**Figure 2:**
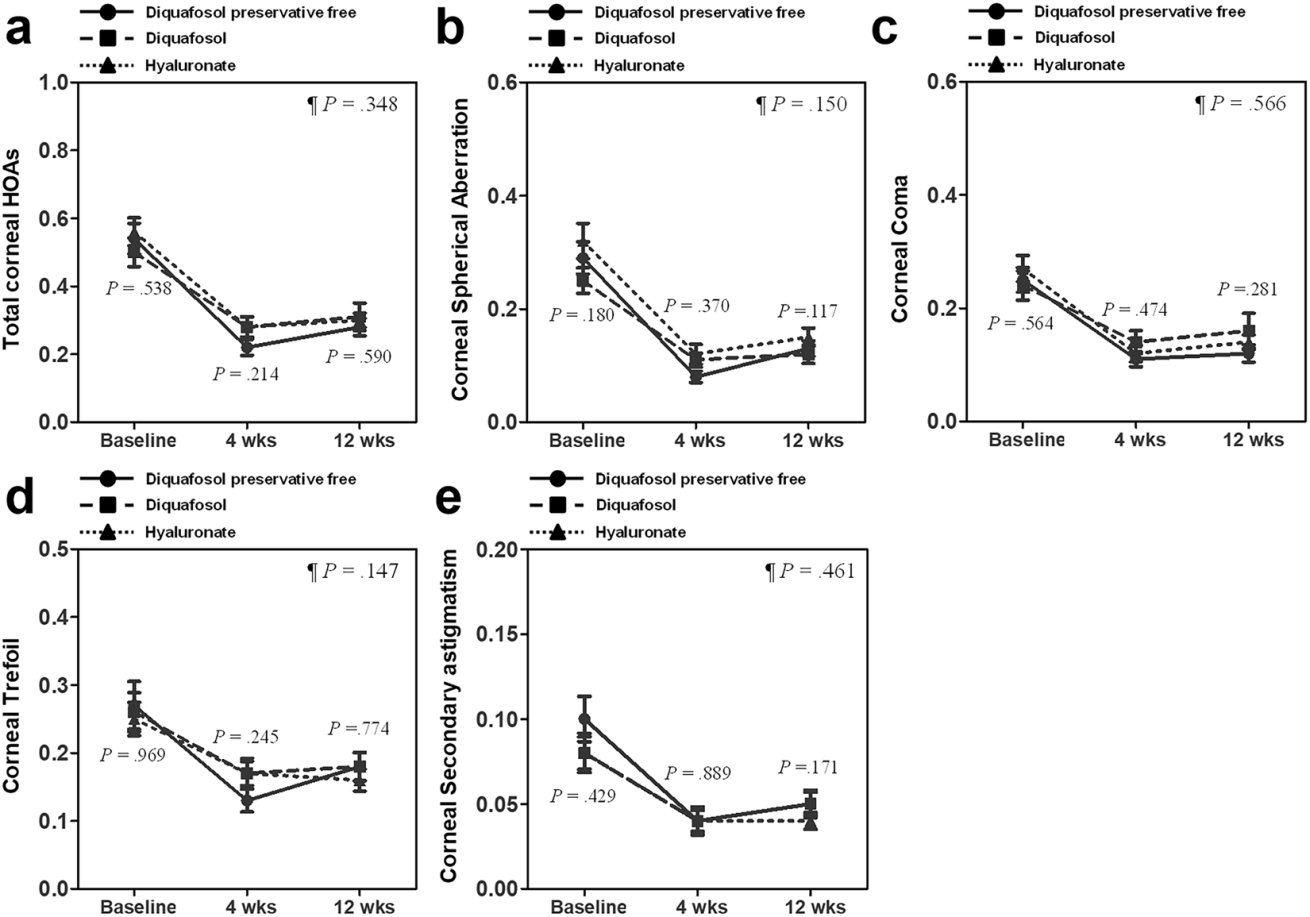
Changes to corneal higher-order aberrations (HOAs) during the treatment period in patients with pre-existing dry eye disease (DED). The mean change to total corneal HOAs **(a)**, spherical aberrations **(b)**, coma **(c)**, trefoil **(d)**, and secondary astigmatisms **(e)** were depicted. Data are presented as mean values ± standard error of the mean (SEM). *P*-values are presented next to all graphs for each study period, indicating the results of statistical analyses among the 3 groups for that period using analysis of variance; ^¶^P-value indicates the overall analysis throughout the study period by generalized estimating equation; ^†^indicates a statistically significant difference between groups 1 and 2 per post hoc analysis; ^‡^indicates a statistically significant difference between groups 1 and 3 per post hoc analysis; ^§^indicates a statistically significant difference between groups 2 and 3 per post hoc analysis.

Preoperative and postoperative higher-order aberrations (HOAs) for the 3 groups are summarized in Table 3. Values for HOAs including total HOAs, spherical aberrations, coma, trefoil, and secondary astigmatism, for all 3 groups were significantly decreased below the preoperative values in all study periods, and there were no significant differences among the 3 groups. Furthermore, no significant differences were noted among the 3 groups in the overall difference analysis throughout the study period (Fig. 2).

**Table 3:** Changes in Corneal Aberrations between Preoperative and 4 and 12 Weeks after Cataract Surgery.

	Group 1 (38 eyes)	Group 2 (41 eyes)	Group 3 (38 eyes)	*P*-value
Total Higher order aberrations				
Preoperative	0.54 ± 0.27	0.50 ± 0.26	0.56 ± 0.25	0.538
4wks	0.22 ± 0.14*	0.28 ± 0.20*	0.28 ± 0.18*	0.214
12wks	0.28 ± 0.15*	0.31 ± 0.25*	0.30 ± 0.13*	0.590
Spherical Aberration				
Preoperative	0.29 ± 0.17	0.25 ± 0.14	0.32 ± 0.19	0.180
4wks	0.08 ± 0.58*	0.11 ± 0.08*	0.12 ± 0.11*	0.370
12wks	0.13 ± 0.08*	0.12 ± 0.10*	0.15 ± 0.10*	0.117
Coma				
Preoperative	0.25 ± 0.13	0.24 ± 0.16	0.27 ± 0.14	0.564
4wks	0.11 ± 0.08*	0.14 ± 0.13*	0.12 ± 0.09*	0.474
12wks	0.12 ± 0.09*	0.16 ± 0.19*	0.14 ± 0.08*	0.281
Trefoil				
Preoperative	0.27 ± 0.21	0.26 ± 0.18	0.25 ± 0.15	0.969
4wks	0.13 ± 0.10*	0.17 ± 0.14*	0.17 ± 0.14*	0.245
12wks	0.18 ± 0.12*	0.18 ± 0.13*	0.16 ± 0.10*	0.774
Secondary astigmatism				
Preoperative	0.10 ± 0.08	0.08 ± 0.06	0.08 ± 0.07	0.429
4wks	0.04 ± 0.05*	0.04 ± 0.04*	0.04 ± 0.04*	0.889
12wks	0.05 ± 0.04*	0.05 ± 0.05*	0.04 ± 0.03*	0.171

Data are presented as mean ± standard deviation. *P*-value indicates the statistical analysis among the three groups. *Indicates a statistically significant result when compared with preoperative value.

## Discussion

The present study investigated the clinical efficacy of preservative-free diquafosol 3% ophthalmic solution compared with preservative-containing diquafosol 3% ophthalmic solution and preservative-free sodium hyaluronate 0.15% ophthalmic solution in pre-existing DED patients who underwent cataract surgery. As reported previously^5,6,10^, diquafosol and hyaluronate treatment improved dry eye symptoms and HOAs in this study. Although corneal HOAs were comparable among the 3 groups, dry eye symptoms were much improved in the diquafosol treatment groups as compared to the hyaluronate treatment group. In addition, though dry-eye and MGD parameters were comparable between the preservative-free and preservative-containing diquafosol groups at 1 month postoperatively, these improvements were most robust in the preservative-free diquafosol group at 3 months after surgery.

According to a meta-analysis of randomized controlled trials comparing the efficacy of diquafosol and artificial tears for the management of DED following phacoemulsification^6^, diquafosol significantly improved multiple outcomes including TBUT, Schirmer test scores, and corneal fluorescein staining scores beyond those obtained from the use of artificial tears. Similarly, Park and colleagues revealed that OSDI was significantly improved in diquafosol-treated individuals^5^.

We found that TBUT was better in diquafosol groups than in hyaluronate group at 1 month after surgery, which implies that diquafosol is more effective than hyaluronic acid in dry eye treatment after cataract surgery, as suggested by the previous studies. Similarly, the postoperative OSDI was better in diquafosol groups than in hyaluronate group. Although the TBUT and OSDI results of the present study were similar to those reported previously, there were no significant differences in the Schirmer test or corneal staining scores among the 3 groups. Wu and colleagues reported that unlike TBUT, Schirmer test scores which evaluate aqueous tear production, did not demonstrate consistent improvements after diquafosol treatment in DED patients^17^, as we also report in the current study. Other studies have also shown that Schirmer test scores did not improve after diquafosol treatment of DED patients following cataract surgery^2,13^. Given the similarity of results between our study and previous studies, we contend that increased mucin secretion with diquafosol treatment plays an important role in tear film stability over aqueous secretion. Notably, corneal staining scores were improved in all the 3 groups, with no significant differences among the 3 groups. Several previous studies have also demonstrated no significant differences in corneal staining scores between diquafosol and artificial tear groups in patients with DED after phacoemulsification^5,18,19^.

Although, few studies have investigated the efficacy of diquafosol for MGD^20–22^, diquafosol has been shown to have beneficial effects on the condition. Some potential mechanisms for this have been explored. P2Y_2_ receptors have been found in the meibomian gland^23^, and previous studies have suggested that P2Y_2_ agonists increase the concentration of intracellular lipids in cultured rabbit meibomian gland cells and in Cu, Zn-superoxide dismutase-1 knockout mice^24,25^. Until now, there have been no studies evaluating the efficacy of diquafosol in treating MGD following cataract surgery. The relationship between cataract surgery and MGD has been examined in many studies, with MGD identified as a key cause of DED following cataract surgery^8,26,27^. Studies have shown that meibomian gland function deterioration has persisted for 3 months postoperatively, suggesting that MGD may lead to persistent dry eye symptoms^26^. In fact, a recent study of our group revealed meibomian gland orifice obstruction and meibomian gland dropout to be associated with persistent dry eye symptoms after cataract surgery^1^. We report here that diquafosol can improve MGD-related parameters more than sodium hyaluronate in DED patients after cataract surgery. These results suggest that diquafosol may be helpful in the treatment of MGD and may further have protective effects against persistent DED following cataract surgery.

Interestingly, TBUT was significantly improved at 3 months after surgery only in group 1, although TBUT at 1 month after surgery was improved in both groups 1 and 2. This difference may be caused due to long-term preservative usage in group 2. Similarly, the reduction in OSDI scores was comparable between group 1 and group 2 at 1 month postoperatively; however, it decreased further in group 1 at 3 months after surgery, despite not reaching a statistically significant value with groups 1 and 2 at 3 months postoperatively. Chronic preservative usage seems to decrease the efficacy of the eye drops in dry eye treatment. It might have caused further reduction of MGD stage in group 1 than in group 2 at 3 months after phacoemulsification; however, MGD stage reduced in both groups 1 and 2 at 1 month postoperatively.

Regarding corneal HOAs after cataract surgery, we found that corneal HOAs improved after cataract surgery across all 3 groups and there were no significant differences among the 3 groups. We hypothesize that because subjects in the present study had preoperative DED, their use of medication after surgery led to HOA improvements in all groups. However, previous studies have shown that diquafosol simply maintained postoperative HOAs, while artificial tears did not prevent deterioration of HOAs^2,5,19^. Another study revealed that diquafosol treatment reduced HOAs as well as corneal staining scores in aqueous-deficient dry eye, suggesting that improvement in superficial punctate keratopathy resulted in HOA reductions^28^. Similarly, we also hypothesize that improvements in corneal staining scores elicited improvements in HOAs in the present study.

Refinement of eye drop ingredients is necessary for improved clinical outcomes. Preservatives in eye drops have previously been implicated in epithelial damage^29^. Preservative-free medications have previously been shown to lead to greater improvements in OSDI, TBUT, corneal staining scores, and Schirmer test scores in patients with DED after phacoemulsification than preservative-containing medications^16^. Furthermore, glaucoma patients who used preservative-free prostaglandin/timolol fixed combinations manifested better results in not only dry eye parameters, but also meibomian gland features than did those who used preserved prostaglandin/timolol fixed combinations^30^. As outlined here, preservative-free diquafosol led to better dry eye and MGD parameters than preservative-containing diquafosol at 3 months after phacoemulsification in the present study. However, the preservative-free diquafosol group showed results comparable to the preserved diquafosol group at 1 month after surgery. There are two potential reasons for this. First, preservatives may not lead to negative clinical effects in the relatively limited, 1-month timespan assessed here. Second, postoperative medications containing preservatives were used for 1 month following surgery in all groups, with potential significant differences not emerging until 3 months.

While the present study offers some intriguing results, it has some limitations that are worth discussing. First, this study was not designed as a blind clinical trial, so there is a risk of bias even though the researcher has evaluated it as fair as possible. Second, the sample size of the present study was relatively small and our study follow-up period of only 3 months was relatively short. However, it is important to note that most changes to the ocular surface following phacoemulsification resolve within a few months^31,32^. Larger sample sizes and more long-term double-blind studies may allow further validation of the present study’s results. Third, we have some concerns about the confounding effect of postoperative use of topical antibiotics and steroid eye drops in the present study’s results. For example, anti-inflammatory medications are especially associated with dry eye symptoms and may improve MGD parameters to some degree^5,33^. Furthermore, the postoperative anti-inflammatory eye drops used in this study contained preservatives. However, all patients in the 3 groups used postoperative antibiotics and steroid medications in the same way; therefore, the differences between the 3 groups in this study are thought to be mainly generated by the eye drops for dry eye.

Despite the limitations, the current study is valuable because it is the first to report the efficacy of preservative-free diquafosol ophthalmic solution in DED patients. Moreover, we comparatively demonstrated clinical outcomes associated with sodium hyaluronate and diquafosol with and without preservatives in patients with DED who underwent phacoemulsification using a randomized clinical trial design. The present study’s findings provide evidence for a novel treatment strategy involving the use of preservative-free diquafosol for the management of eye health and DED following cataract surgery.

In conclusion, the present study revealed that a 3% diquafosol ophthalmic solution was more effective than a sodium hyaluronate ophthalmic solution in managing DED after cataract surgery, whether it contained a preservative or not. Furthermore, although preservative-containing diquafosol led to clinical outcomes comparable with preservative-free diquafosol at 1 month after surgery, use of preservative-free diquafosol resulted in better outcomes at 3 months after surgery, suggesting that preservative use may be detrimental to DED treatment.

## Patients and Methods

### Subjects

This study involved a prospective randomized controlled clinical trial design and was approved by the Institutional Review Board of Yonsei University College of Medicine (Seoul, South Korea; IRB No. 4-2017-0601; Date of approval: 15 August 2017) and was registered at ClinicalTrials.gov (NCT03640351, Date of registration: 21 August 2018). All study procedures followed the tenets of the Declaration of Helsinki, and written informed consent was obtained from all participants. The study used a prospective randomized design and was conducted according to the original protocol (full protocol available on request).

We enrolled patients with pre-existing DED scheduled to undergo cataract surgery. We defined DED with minor modifications based on the standards set in the Tear Film and Ocular Surface Society Dry Eye Workshop II Diagnostic Methodology report^34^. We diagnosed DED when OSDI score was 13 or more and one of the following two conditions was satisfied: 1) TBUT less than 10 seconds or 2) ocular surface staining showing >5 corneal spots. Participant exclusion criteria included an age less than 20 years, previous use of tetracycline or eye drops except artificial tears within 3 months prior to cataract surgery, the presence of any severe ocular surface disease and/or corneal epithelial pathologies (except DED), a history of previous ocular surgery or trauma, ocular comorbidities, such as glaucoma, uveitis, and cystoid macular oedema. All patients underwent cataract extraction with phacoemulsification and intraocular lens implantation at Yonsei University College of Medicine, Seoul, Republic of Korea, between August 2017 and September 2018. Participants were randomly assigned into the preservative-free diquafosol group (group 1), the preservative-containing diquafosol group (group 2), or the hyaluronate group (group 3) via a simple, unrestricted randomization method by the controller (Supplementary Fig. S1). Group 1 used preservative-free 3% diquafosol tetrasodium ophthalmic solution (Diquas-S; Santen Pharmaceutical Co, Ltd, Osaka, Japan) 6 times a day, while group 2 used 3% diquafosol tetrasodium ophthalmic solution (Diquas; Santen Pharmaceutical Co, Ltd) which contains chlorhexidine as preservative, 6 times a day, and group 3 used preservative-free 0.15% sodium hyaluronate ophthalmic solution (New Hyaluni 0.15%; Taejoon, Seoul, Korea) 6 times a day. All 3 groups used eye drops from postoperative day 1 to 12 weeks after surgery. The calculated total sample size was 111 when we set effect size 0.3, power 0.80, and α 0.05; therefore, we enrolled 50 subjects in each group considering follow up loss. Right or left eye data were randomly chosen using randomization tables, regardless of ocular dominance, refraction, or presence of aberrations.

### Preoperative and postoperative assessments

All subjects underwent dry eye and MGD examinations before and 1 and 3 months after cataract surgery. The subjects did not use any eyedrops prior to 2 hours of examination. These examination parameters included TBUT, type I Schirmer test scores, Oxford staining scores, and OSDI scores. MGD parameters consisted of LLT, lid margin abnormalities, meibum quality, meibum expressibility, MGD stage, and meibomian gland dropout. We evaluated LLT, meibomian gland dropout, TBUT, Oxford staining scores, lid margin abnormality, meibum quality, meibum expressibility, MGD stage, OSDI scores, and Schirmer test scores in order. Corneal aberrations in all subjects were measured via iTrace (Tracey Technology, Houston, TX, USA), as previously described^35^.

TBUT was evaluated by placing a single fluorescein strip (Haag-Streit, Koeniz, Switzerland) over the inferior meniscus. The mean of 3 evaluation attempts was used for analysis. The Schirmer test was performed without topical anaesthesia using a standard paper strip (Eagle Vision, Memphis, TN) for 5 minutes. The OSDI Questionnaire, developed by the Outcomes Research Group at Allergan (Irvine, CA), was used to assess dry eye symptoms. LLT was measured with the LipiView 2 system (Tear-Science, Morrisville, NY). Meibomian gland dropout was graded between 0 and 4 from a low lid meibomian gland image obtained with the LipiView 2 system^36^. Lid margin abnormalities were graded from 0 to 4 according to the following 4 factors: vascular engorgement, plugged meibomian gland orifice, anterior or posterior displacement of the mucocutaneous junction, and irregularity of the lid margin^1,37,38^. The meibum quality of 8 glands in the centre of the lower lid was scored from 0 to 24^1,38^. Meibum expressibility was assessed using firm digital pressure over 5 lower lid glands and calculated semi-quantitatively, as previously described^1,39^. The MGD stage was determined by evaluating clinical symptoms, fluorescein staining of the cornea and conjunctiva, lid margin abnormalities, expressibility, and any altered secretion by a single clinician (I.J.) as previously described^1,40^.

### Surgical technique

All cataract surgeries were performed by one surgeon (T.K.). Phacoemulsification and cataract extractions were performed using the Centurion Vision System (Alcon Laboratories, Inc., Lake Forest, CA, USA) after a 2.8-mm clear corneal incision was made under topical anaesthesia. All patients underwent intraocular lens implantation in the capsular bag. Patients were instructed to instil one drop each of topical 0.5% levofloxacin (Cravit, Santen Pharmaceutical, Osaka, Japan; preservative-free) and 0.1% fluorometholone (Ocumetholone, Samil Pharmaceutical, Seoul, Korea; containing benzalkonium chloride as preservative) 4 times a day, and 0.1% bromfenac sodium (Bronuck, Taejoon, Seoul, Korea; containing benzalkonium chloride as preservative) 2 times a day for 4 weeks following surgery.

### Statistical analyses

Data are expressed as mean values ± standard deviation or standard error as described in the legend of the figures and tables. Analyses of variance, followed by Tukey’s multiple comparison test or the Kruskal-Wallis test with Bonferroni post hoc analysis, were performed, as appropriate. Differences were considered to be statistically significant at *P* < 0.05. A generalized estimating equation with Bonferroni post hoc analysis was used for repeated measurements to compare overall differences throughout the study period among the 3 groups. For Bonferroni post hoc analyses, P < 0.0167 was considered significant. Statistical analyses were performed using SPSS statistics software (version 23; IBM Corporation, Armonk, NY).

## Supplementary information

Supplementary information

## Data Availability

The datasets generated during and/or analysed during the current study are available from the corresponding author on reasonable request.
