# Supplementary material for: Effects of Preservative-free 3% Diquafosol in Patients with Pre-existing Dry Eye Disease after Cataract Surgery: A Randomized Clinical Trial
Source: Sci Rep. 2019 Sep 2;9:12659. doi: 10.1038/s41598-019-49159-0 (PMC6718409; doi:10.1038/s41598-019-49159-0)
Supplement: Supplementary file 1 — Supplementary information [file 41598_2019_49159_MOESM1_ESM.pdf]

# Effects of Preservative-free 3% Diquafosol in Patients with Preexisting Dry Eye after Cataract Surgery: A Randomized Clinical Trial

Ikhyun Jun, MD, PhD[1,2], Seonghee Choi, MD[1], Geun Young Lee, MD[1], Young Joon Choi, MD[1], Hyung Keun Lee, MD[1,2], Eung Kweon Kim, MD[1,2], Kyoung Yul Seo, MD[1], Tae-im Kim, MD[1,2]

[1]The Institute of Vision Research, Department of Ophthalmology, Yonsei University College of Medicine, 50-1 Yonsei-ro, Seodaemungu, Seoul 03722, Korea

[2]Corneal Dystrophy Research Institute, Department of Ophthalmology, Yonsei University College of Medicine, 50-1 Yonsei-ro, Seodaemungu, Seoul 03722, Korea

**Supplementary Figure S1.**

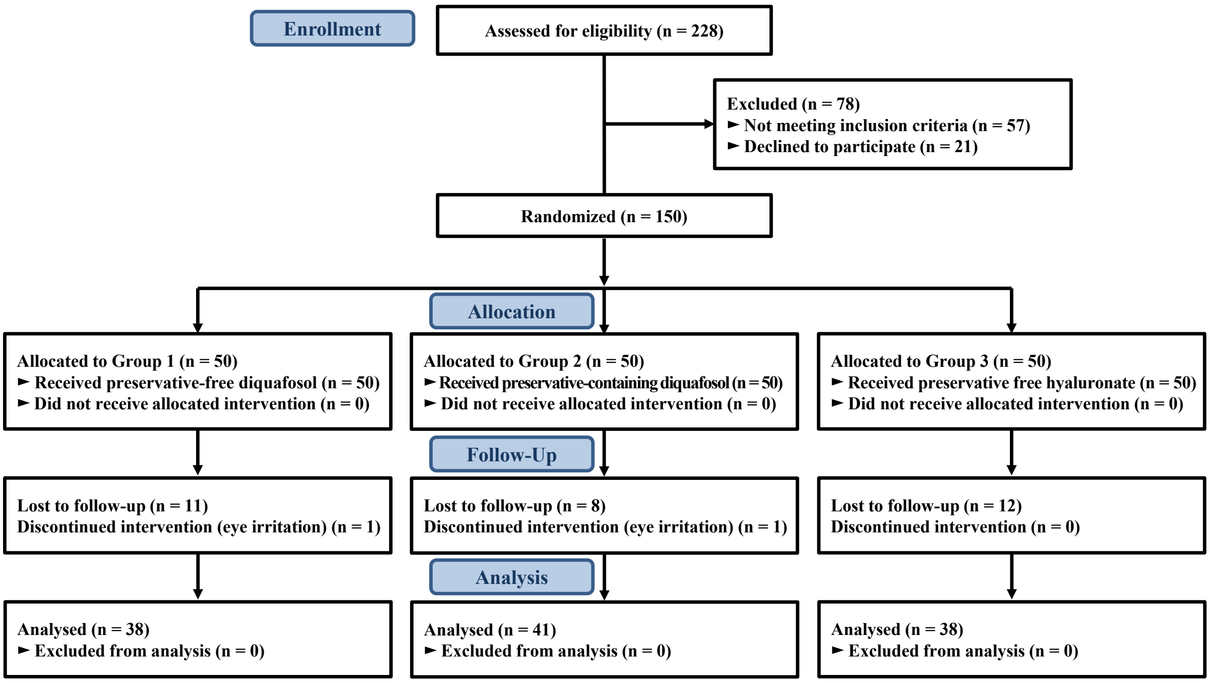


**Supplementary Figure S1.** Consolidated Standards of Reporting Trials (CONSORT) flow diagram illustrating the randomization procedure used in the present study.
